# Quality of Set Yogurts Made from Raw Milk and Processed Milk Supplemented with Enriched Milk Fat Globule Membrane in a Two-Stage Homogenization Process

**DOI:** 10.3390/foods10071534

**Published:** 2021-07-02

**Authors:** Joshua Oladapo Ibitoye, Binh Ly-Nguyen, Duy Nghia Le, Koen Dewettinck, Antoine P. Trzcinski, Thi Thanh Que Phan

**Affiliations:** 1 Department of Food Technology, Can Tho University, Can Tho City 900000, Vietnam; successibt@gmail.com (J.O.I.); lnbinh@ctu.edu.vn (B.L.-N.); ldnghia@ctu.edu.vn (D.N.L.); 2 Laboratory of Food Technology and Engineering, Department of Food Safety and Food Quality, Ghent University, Coupure Links 653, B-9000 Ghent, Belgium; koen.dewettinck@ugent.be; 3 School of Civil Engineering and Surveying, University of Southern Queensland, West Street, Toowoomba, QLD 4350, Australia; antoine.trzcinski@usq.edu.au

**Keywords:** milk fat globule membrane, two-stage pressure homogenization, milk proteins, processed milk, raw milk, set yogurts

## Abstract

Dairy products are relevant in the food industries as functional ingredients for several food products and contribute towards human nutrition in ameliorating certain disorders. In this study, set yogurts were produced from raw milk and processed milk combined with 4% Lacprodan^®^PL20 concentration and subjected to two-stage pressure homogenization. The total solids concentration of the mixture was raised to 15% using SMP (skim milk powder). The purpose of this study was to investigate the effect of Lacprodan^®^PL20 on the set yogurt quality produced by homogenization-induced pressure and its interaction with milk components. The changes in the physical and chemical attributes of the milk fat globule membrane (MFGM) via destabilization of the membrane significantly affected the physicochemical properties of set yogurts produced from processed or raw milk. There was a slight variation in MFGM-specific proteins detected in the set yogurts. Set yogurt produced from homogenized raw milk (HRM) had a considerably higher water-holding capacity, firmness, and apparent viscosity. The microstructure of HRM was dense and compacted, unlike non-homogenized raw milk (NRM) with large MFGM fragments and pore holes between the matrixes. The inclusion of homogenization showed a remarkable improvement in set yogurt quality, promoting interaction between MFGM components and milk proteins.

## 1. Introduction

The biological membrane surrounding fat globules in milk is known as the milk fat globule membrane (MFGM); these globules are involved in many biological functions and interactions with the surrounding milk proteins. The MFGM primarily contains specific proteins that are bound to the carbohydrate and phospholipid bilayers with a diameter between 0.1 µm and 15 µm [1,2,3]. These components are amphiphilic and actively involved in the changes that occur in the structure of set yogurts. The bilayer of polar lipids consists of zwitterionic sphingomyelin (SM), phosphatidylcholine (PC), and phosphatidylethanolamine (PE) predominantly at the surface of the membrane, and anionic phosphatidylinositol (PI) and phosphatidylserine (PS) at the inner part, which are proactively linked with proteins [2,4,5] The specific proteins in the MFGM interact with other proteins, phospholipids, and/or milk proteins by creating a disulfide bridge. For instance, PAS 6/7 (lactedherin) binds with phospholipids as reported by Fortunato et al. [6]. Butyrophilin (BTN), xanthine dehydrogenase, and perilipin-2 (ADFP) were shown to be involved in the bonding process of MFG to milk, with adipophilin having a strong affinity for triglycerides [7]. Both butyrophilin and xanthine oxidase (XO) are tightly attached to some fatty acids such as palmitic, stearic, and oleic acids [8,9].

Processing could unilaterally affect the MFGM with several research studies indicating some changes in the structure, interaction, and nutritive values, along with rheological, physical, and chemical attributes such as protein loading, ionic concentration, polar lipid contents, water-holding capacity, MFGM size, solubility, and affinity strength, among others [4,10,11,12]. These modifications could sway consumer perceptions, leading consumers to accept or reject the products depending on their taste, appearance, flavor, and texture. In short, homogenization induces significant changes in the MFGM that positively affect its interaction with other materials. Crucial evidence suggests that homogenization plays a role in the size reduction, membrane destabilization, and surface interaction of MFGM proteins and non-MFGM proteins (e.g., milk casein and whey proteins) [13,14]. Comparative studies carried out between homogenized and non-homogenized MFGM revealed some changes in the structure and adsorption of MFGM, which enhanced the product attributes [1,15,16]. Recently, homogenization of MFGs during the production of milk powder resulted in an increase in the zeta potential and a reduction in size, which in turn, increased surface attraction [2]. Moreover, heat treatment of milk and the MFGM showed modification of some physical attributes associated with set yogurts; heating milk at 60–65 °C was found to link β-lactoglobulin with the MFGM, which is lower than the denaturation temperature of β-lactoglobulin (78 °C) [12,17,18]. It was suggested that β-Lactoglobulin may bind to the MFGM via sulfhydryl-disulfide interchange reactions [19]. Little amounts of κ-casein also appeared to interact with MFGM when heating whole milk above 80 °C [9,18].

However, to date, no further studies aimed at improving quality have elucidated possible changes induced by a two-stage pressure homogenization process of the MFGM incorporated into set yogurt produced from raw milk or processed milk, leaving the profound changes that occur as the MFGM interacts with milk fractions unreported. As part of this study, processed milk and raw milk were used to deeply probe some differences in set yogurt quality in terms of MFGM interaction and distribution as well as the contribution towards quality improvement.

## 2. Materials and Methods

### 2.1. Materials

Raw milk collection was carried out on a farm in the Mekong Delta, Vietnam; raw milk with pH 6.7 was quickly stored in aseptically clean containers at 4 °C in a refrigerator for a few hours to avoid changes in the quality. The processed milk was a finished product of a company in Australia. Skim milk powder (SMP) and a yogurt starter culture were obtained from Asia Sai Gon Food Ingredients, Ho Chi Minh City, Vietnam. The starter culture consisted of *Lactobacillus delbrueckii* subsp. *bulgaricus* and *Streptococcus thermophilus* (ratio 1:1); this was stored below −18 °C. Arla Foods (Viby, Denmark) supplied Lacprodan^®^PL20, an enriched MFGM in the form of a spray-dried powder rich in milk phospholipids and proteins. This product has a registered trademark. Enriched MFGM material was obtained during butter oil production (40% fat) using centrifugation and membrane filtration processes. Thereafter, the final concentrate, comprising over 20% phospholipids in total solids, was spray-dried. The Lacprodan^®^PL20 production procedure is patent-pending WO2006/128465 A1 (Arla Foods, Viby J, Denmark).

#### Set Yogurt Preparation

**Figure 1:**
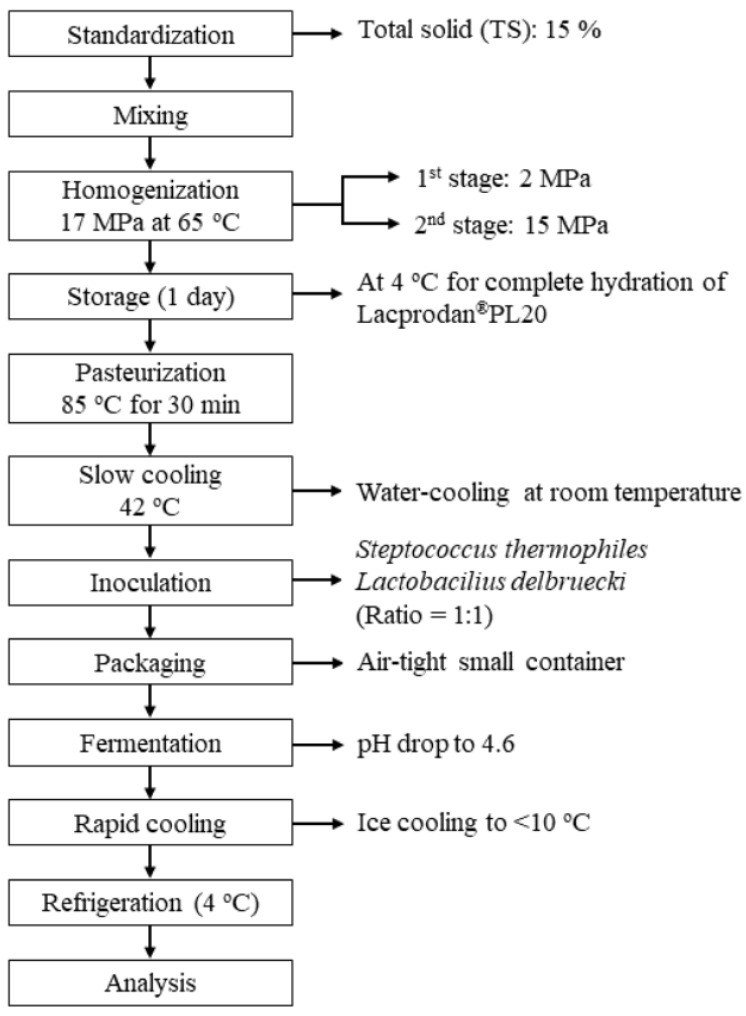
Schematic diagram of set yogurt preparation using two-stage homogenization.

Set yogurts were produced from raw or processed milk supplemented with 4% Lacprodan^®^PL20 (w/v) and fermented with 0.05% *Streptococcus thermophilus* and *Lactobacillus delbrueckii* subsp. *bulgaricus*. The experiment was replicated thrice. The raw and processed milk samples were subjected to homogenization after adding 4% Lacprodan^®^PL20, though not the control samples. The samples were heated in a container to 65 °C and thoroughly mixed for 1 min at 1000 rpm, homogenized at 17 MPa (1st stage = 2 MPa; 2nd stage = 15 MPa) in a two-stage homogenizer (Homolab 2.20, Parma-Italy), and then stored overnight at 4 °C in a refrigerator for complete hydration. Thereafter, the samples were pasteurized at 85 °C for 30 min in a water bath and cooled to 42 °C before inoculation. The fermentation process was terminated as soon as the pH level reached 4.6. The set yogurts were cooled with ice water and stored at 4 °C for one day before the analysis was performed. The successive steps are outlined in Figure 1.

### 2.2. Methods

#### 2.2.1. Physicochemical Analyses

The dry matter content was gravimetrically determined after oven-drying to a constant weight at 105 °C. The total protein content of the set yogurts was determined by the Kjeldahl method using 6.38 as the conversion factor. The total lipid content was determined using the Röse-Gottlieb method [20]. The ash content was determined by ashing in a furnace at 550 °C. The lactose content of the set yogurts was determined by subtracting the protein, ash, and lipid contents from the dry matter. Dry matter, protein, and ash were analyzed using AOAC and Arslan and Bayrakci methods [21,22]. All chemical analyses were repeated thrice. The measurement of total acidity was done according to the method described by Bradley et al. [23]. pH readings were taken every hour via the Logger lite application using a pH meter (Hanna, H12210, Parma, Italy).

#### 2.2.2. Specific Protein Determination

The protein separation profile and all the reagents used were obtained from Invitrogen (Merelbeke, Belgium). The sample preparation was in accordance with Le et al. [24]. The wet gel was scanned at 400 dpi with a high-resolution xv transmission scanner (UMAX Powerlook III, Taipei, Taiwan). Protein separation was done on gradient (4–12%) polyacrylamide gels with an Xcell Surelock system. The main proteins were named in accordance with Mather [25]: XO, CD36, BSA, BTN, PAS 6/7, ADPH.

#### 2.2.3. Firmness

Firmness assessment was carried out after fermentation and storage in a refrigerator at 4 °C for a day. The Brookfield texture analyzer manufactured by Brookfield Engineering Laboratories was used to measure the firmness using the probe TA 48. A compression test with a maximum load of 10,000 g and depth of 10 mm penetration was applied to determine the firmness. The temperature was kept constant at 5 °C during the test [26]. The firmness assessment was carried out in duplicate.

#### 2.2.4. Apparent Viscosity

The set yogurts were gently stirred a few times in a clockwise direction with a spoon prior to viscosity measurements as described by El-Sayed et al. [27] with slight modifications. The samples were poured into a cup (height 160 mm x diameter 60 mm) and subjected to a constant shear rate of 0.5 s^−1^ for 150 s in a DV-E viscometer (Brookfield Engineering, Middleborough, MA, USA) equipped with the T-piece spindle-4 to measure the viscosity at 5 °C. A visual recording device was used to take a reading every 15 s in centipoise (cp), and finally, the readings were converted to Pascal-seconds (Pa.s) [28]. This method was repeated twice.

#### 2.2.5. Determination of Water-Holding Capacity (WHC)

The WHCs of set yogurts were measured at 5000 rpm using a centrifuge (Hermle, Z323K, Wehingen, Germany); this was repeated thrice. For each sample, 25 g was weighed into a falcon plastic tube, then capped and centrifuged for 25 min at 5 °C [26]. The whey was carefully removed, weighed and the WHC was calculated as follows;

WHC (%) = [(Sample weight − Whey dispelled)/Sample weight] × 100

#### 2.2.6. Microstructure Observation

A scanning electron microscope (SEM, JSM 5500) was used to examine the microscopic structure of the set yogurts after incubation with little adjustment. The set yogurts were freeze-dried at −96 °C for 10 h before examination. The SEM photomicrograph parameters were 20 µm scale bar and 900 times magnification.

#### 2.2.7. Statistical Analysis

The experimental data were analyzed using one-way analysis of variance (ANOVA) and significant differences among the means were determined at a 95% confidence level using the software package MINITAB for Windows 10 (Minitab Inc., State College, PA, USA, 2010). Tukey’s test was used to determine the paired comparison of the mean when a significant difference was observed at *p* < 0.05.

## 3. Results and Discussion

Homogenization induced changes in the compositional characterization of set yogurts produced from raw milk or processed milk combined with an enriched MFGM.

The proteins and fat contents of raw milk and processed milk were adjusted to 3.3% and 3.67%, respectively, and the total solids of the milk samples were within 12%.

The assessment of homogenization of the chemical composition of set yogurts formulated with Lacprodan^®^PL20 showed a significant difference, as shown in Table 1, with a *p*-value < 0.05. The protein contents of set yogurts produced from homogenized processed milk (HPM) or raw milk (HRM) were slightly similar, with 30.48 ± 1.33 or 33.90 ± 2.88 g/100 g dry matter, respectively, and significantly lower than the non-homogenized processed milk (NPM) and raw milk (NRM). The fat content of homogenized raw milk (HRM) was statistically different than homogenized processed milk (HPM), which was similar to NPM and NRM. The ash contents were closely related to NPM, with the highest value of 7.19 ± 0.18 g/100 g dry matter. The lactose content of NRM exhibited similarity to NPM and HRM, but not HPM, with 39.64 ± 1.10 g/100 g dry matter. The total solids were similar, with slight differences among them. NRM was completely different from NPM in terms of the total solids (non-fats) content. Also, an increase in total solids after homogenization was observed. The application of pressure homogenization affected the chemical composition characteristics of MFGM, as seen in the results, which is consistent with past studies [2,3]. The homogenization process plays a significant role in the disruption of the membrane, size reduction, increase in the surface area, and adsorption of more caseins at the MFGM interface [29]; however, two-stage homogenization prevented clustering of the MFGM for maximum interaction with milk proteins. The disintegration of the MFGM by pressure homogenization led to more adsorption of casein [30]. Heat treatment accounted for the interaction of denatured *β*-LG and *α*-LA, which failed to interact with κ-CN during homogenization at the interface [10], thus reducing the protein content of set yogurts from homogenized raw milk and processed milk (HRM and HPM) compared to non-homogenized samples (NPM and NRM) [29]. As the size of the MFGM reduced and its surface area increased, the tendency for there to be an interaction between phospholipids and milk proteins became extremely high due to MFGM polarity, which considerably influenced its affinity. The result showed that using processed milk (pasteurized product) with a standardized composition caused some changes to the chemical composition of set yogurts, without alteration to the functionality of the MFGM due to the denaturation of milk proteins [31,32].

**Figure 2:**
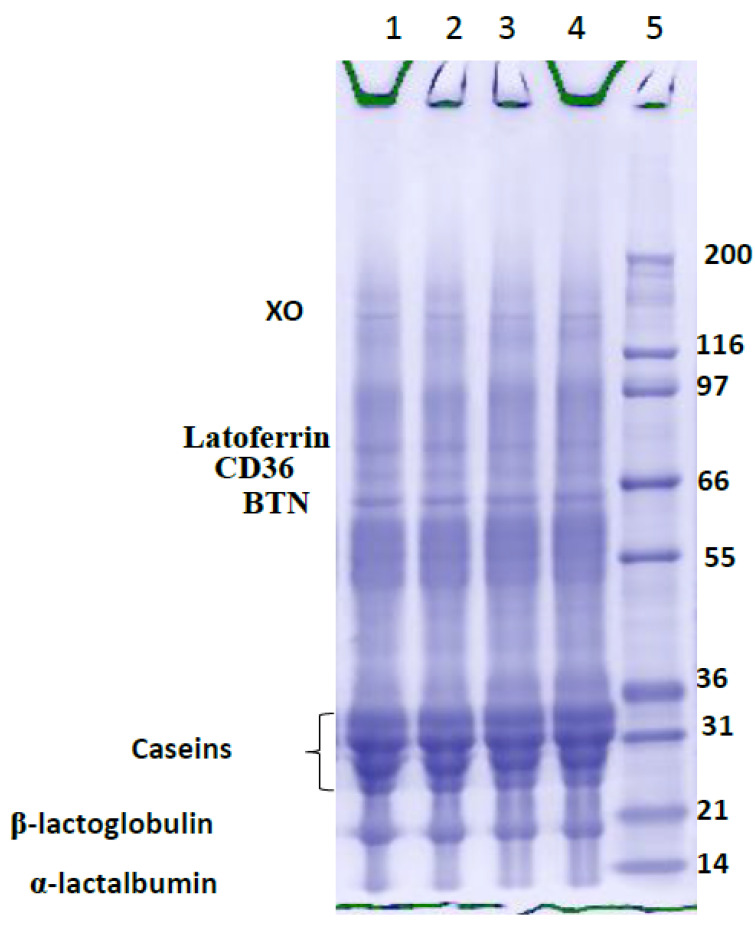
SDS-PAGE elucidates the MFGM-specific protein profile of set yogurts prepared from different milk materials with a 4% enriched MFGM fragment under two-stage pressure homogenization. Line 1: Non-homogenized raw milk (NRM), Line 2: Homogenized raw milk (HRM), Line 3: Homogenized processed milk (HPM), Line 4: Non-homogenized processed milk (NPM), Line 5: SDS-PAGE molecular weight standard.

Further elucidation of the chemical composition with regards to the influence of pressure homogenization on the MFGM incorporated into set yogurt led to the identification of several MFGM-specific proteins using Sodium Dodecyl Sulphate-Polyacrylamide Gel Electrophoresis (SDS-PAGE) techniques stained with Coomassie blue. The SDS-PAGE image in Figure 2 points to the presence of some MFGM proteins: butyrophilin (BTN), cluster of differentiation (CD36), xanthine oxidase (XO), periodic acid Schiff V/VI (PAS 5/6), adipophilin (ADPH), and milk proteins (casein, *β*-LG, and *α*-LA) including lactoferrin (globular protein), which were properly stained due to the abundance of basic amino acids with a higher degree of Coomassie blue binding, while the glycoproteins were not properly revealed due to their carbohydrate moieties, which prevented the binding of Coomassie blue [33,34] (these are only detected by PAS or silver staining). Heating milk at a high temperature almost completely depleted the peripheral proteins PAS 7 and PAS 6 [18]; this could be a result of casein micelles adsorbed to the MFGM covering PAS 6 and PAS 7 through spreading, and thus protecting them from loss during subsequent heating [14]. These two reasons can explain the difference in the protein contents of HPM and HRM. In another study conducted by Sharma et al. [35], heating milk to 55 °C decreased the activity of native enzymes (alkaline phosphatase, XO), and at 60 °C, MFGM-specific proteins (BTN and XO) aggregated via intermolecular disulfide bonds. This explains the low amount of protein content in set yogurts produced from processed milk with an enriched MFGM material (NPM and HPM). Despite applying the homogenization process, which contributed massively towards structural attributes, set yogurt with an MFGM might contain enormous nutritional and nutraceutical values, promoting good human health. For instance, phosphatidylcholine and sphingomyelin promote metabolism and membrane construction in the brain and nerves [36], and specific proteins possess bioactive properties like antibacterial activity [37,38], cancer-inhibiting effects [39], and prevention of autoimmune conditions, such as multiple sclerosis [40] or autism [41].

### 3.1. PH and Total Acidity

**Figure 3:**
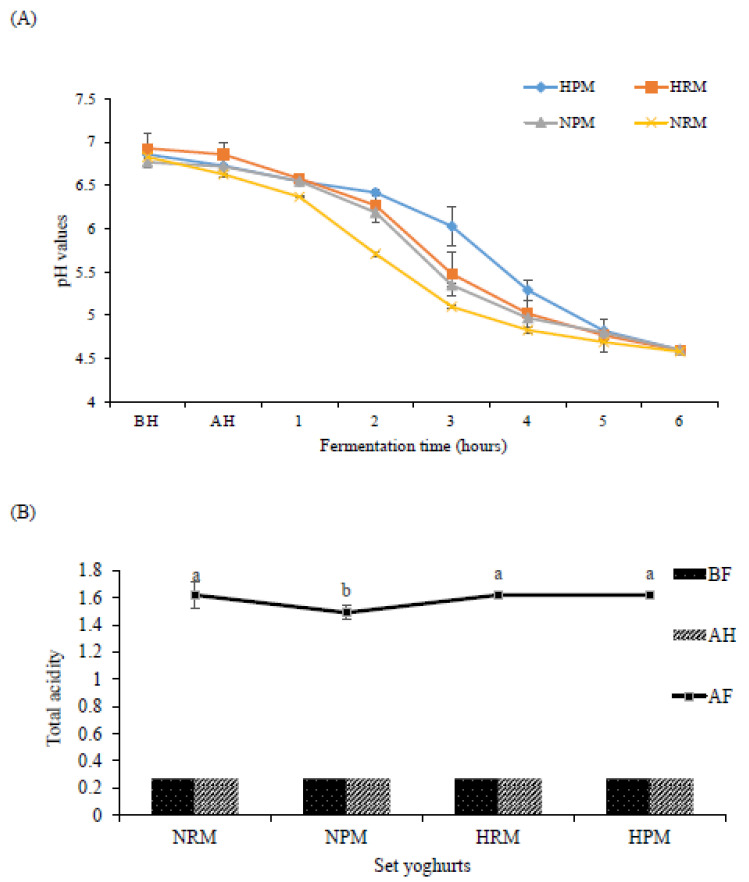
pH values (**A**) and total acidities (**B**) of set yogurts enriched with MFGM produced from raw milk or processed milk under pressure homogenization. BF: Before fermentation; AF: After fermentation; AH: After heating. NPM: Non-homogenized processed milk, NRM: Non-homogenized raw milk, HRM: Homogenized raw milk, HPM: Homogenized processed milk. a, b represent the significant differences in the total acidity among set yoghurts. The total acidity is expressed as % lactic acid.

Certain studies have shown some changes in the zeta-potential of MFGM, which is made of protein and other surface-active components, especially when homogenization is applied [2,42]. Some of these changes include an increase in the net charge of the solution resulting in a 20% concentration increment [43]. The main phospholipids present in the MFGM are zwitterionic sphingomyelin (SM), phosphatidylcholine (PC), and phosphatidylethanolamine (PE), with less abundant anionic phosphatidylserine (PS) and phosphatidylinositol (PI) [9,44], while the main proteins are also polar [45]. The results obtained in this study are in line with a previous study conducted by Le et al. [26]. The exposure of anionic PI and PS head-groups by homogenization coupled with the proteins carrying a negative zeta potential accounted for the increase in the ionic strength of the set yogurts. Invariably, the MFGM has the potential to increase the pH values as seen in the result obtained (Figure 3A). With the application of pressure homogenization, the pH was higher due to MFGM surface exposure and disruption. After heating (AH), the pH steadily decreased as a result of the deposition of milk proteins, in particular casein and whey proteins, on the surface of the MFGM fragments [18,46]. The curve of HPM between 2–5 h of fermentation was above others before lowering, whereas the pH values of NRM were low, showing a huge impact of homogenization with material differences resulting (Figure 3A). The curve of HPM between 2–5 h of fermentation was above others before lowering, whereas the pH values of NRM were low, showing a huge impact of homogenization on the type of milk (Figure 3A). During fermentation, *Lactobacillus delbrueckii* subsp. *bulgaricus* and *Streptococcus thermophilus* quickly converted lactose into lactic acids, resulting in the termination of the fermentation process as soon as the pH values were reduced to 4.6 (after 6 h). Lacprodan^®^PL20 showed no elongation of the fermentation time beyond 6 h, as found in Figure 3A.

The experimental result of total acidity (TA) for set yogurts supplemented with Lacprodan^®^PL20 (enriched MFGM) after fermentation (AF) showed a statistically significant difference, with NPM having the lowest value at *p* < 0.05. The total acidity before and after heating (BH and AH) remained constant, as total acidity was calculated based on lactic acid production (Figure 3B). The reduction of MFGM fragment size through homogenization triggered the release of more monosaccharides from the carbohydrate moieties of the MFGM components, serving as an energy source for lactic acid bacteria to convert more lactose into lactic acid during the fermentation process [47,48]. The lactic acid in NRM, HRM, and HPM was the same. Homogenization had little or no impact in this case; the only differences were the impact of pasteurization before homogenization [49] and probably the lactose content in the set yogurts (NPM had the lowest value in Table 1).

### 3.2. Water-Holding Capacity

**Figure 4:**
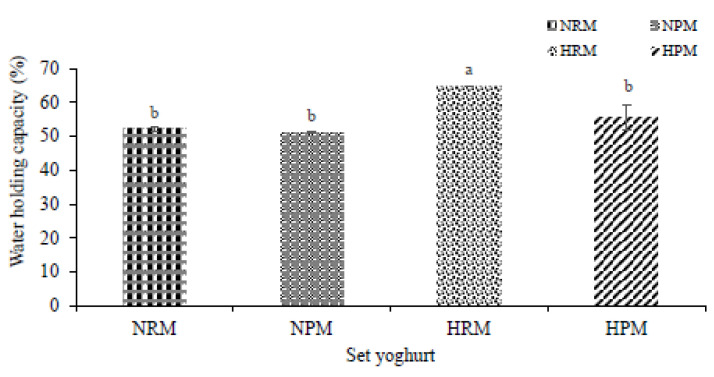
Homogenization-induced substantial changes to water-holding capacity of set yogurt produced from raw milk or processed milk combined with an enriched MFGM. a, b represent the significant differences in the total acidity among set yoghurts.

The water-holding capacities of NPM, HPM, and NRM were significantly different from HRM at *p* < 0.05, as shown in Figure 4. MFGM contains polar components (specific proteins and phospholipids) with the ability to absorb and hold water within a system [50,51]. Fundamentally, the results proved that homogenization enhanced the polarity of MFGM as it exposed the surfaces of the fragments with more anionic charges (hydrophilic heads), in order to attract more water, especially in HRM. The explicit exposure of the surface of polar heads by homogenization directly contributed to the functionality of MFGM for the water-holding capacity of the system, which directly improved the quality of the set yogurt. These physical and chemical heterogeneities of polar lipids occurring in the MFGM could govern the lipid-protein interactions at the surface [52], hence providing the specific functional attributes of hydrophilic attraction. As pressure homogenization impacted the membrane, syneresis drastically reduced, as was observed at the surface of the set yogurt produced from homogenized raw milk, with enriched MFGM material (HRM). In a similar study performed using MFGM material, buttermilk material, and skimmed milk (up to 4%) to produce set yogurt, the water-holding capacity of set yogurt with MFGM possessed the highest values, and at the same time, showed a reduction of whey at the surface compared to other materials [26]. Despite the fact that MFGM improved the WHC in this study, the water-holding capacity was still low compared to when homogenization was applied (Figure 4). Conversely, homogenization carried out after pasteurization led to unchanged WHC (HPM) because the whey proteins were denatured and complexed with caseins, therefore, reducing the level of interaction with the enriched MFGM [31].

Consumers are concerned about questionable additives and/or ingredients, considering their long-term effects on health. As a result, many consumers are advocating for clean-label foods so as to mitigate any health concerns. The use of MFGM combined with a technological process would go a long way to improving the quality of set yogurt (structural and physical properties) without adding any questionable stabilizers.

### 3.3. Firmness of Different Set Yogurts with an Enriched MFGM

The results listed in Table 2 indicate that tangible differences existed in the non-homogenized and homogenized raw milk (NRM and HRM) when compared to non-homogenized and homogenized processed milk (NPM and HPM). Homogenization slightly increased the firmness of set yogurts (HRM and HPM). During the homogenization of milk, the size of the fat globules reduces, which in turn, increases the surface area of the fat globules, including MFGM, by attracting more amphiphilic molecules to the active surface [53,54,55], thereby promoting the texture of set yogurts. Moreover, there was a possibility of protein-protein interaction, which further strengthened the gel strength during homogenization. However, instead of having many native MFGM fragments that could serve as structure breakers or inert fillers, homogenization reduced the sizes and volumes by activating the surface area for close molecular interaction. On the other hand, milk pasteurization before homogenization had no statistically substantial effect on the firmness of set yogurt. Notwithstanding, the firmness of homogenized processed milk (HPM) was slightly raised, showing a level of interaction with no statistical variation at *p* < 0.05. Singh [9] discussed the influence of heat treatment on MFGM protein interaction with the milk proteins, and reported that MFGM proteins usually interact with milk proteins at an optimum temperature, and any rise in temperature above that would definitely impair their functionality.

### 3.4. Variation in the Apparent Viscosity

**Figure 5:**
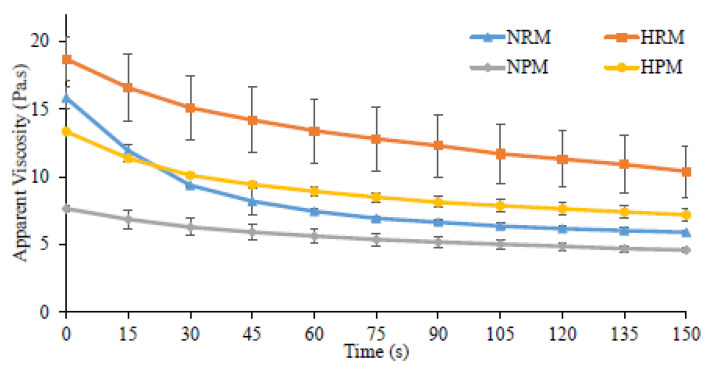
Influence of two-stage pressure homogenization on Lacprodan^®^PL20 as it affects the apparent viscosity of set yogurts produced from raw milk and processed milk.

The curve of apparent viscosity for set yogurts showed a shear-thinning behavior as time increased at a constant shear rate of 0.5 s^−1^ (Figure 5). The initial and final viscosities of set yogurts with the application of pressure homogenization [29] were higher than non-homogenized samples with statistically significant differences (Table 3). Also, homogenization had a substantial effect on structural loss. The non-homogenized raw milk (NRM), with 62.7% structural loss, experienced fast dissociation, probably because the interaction was not strong enough to withstand the high shear stress, which quickly removed proteins and phospholipids from the MFGM surface and caused fat globule coalescence [56,57]. Furthermore, the sporadic chemical and structural heterogeneities in the polar lipid composition may be involved in the modulation of many membrane functions, in particular, the interactions with milk proteins. According to Holzmüller and Kulozik [58], strong binding of PAS 6/7 to MFGM fragments rich in PC and SM occurred during the churning of cream and disruption of the MFGM at high shear forces. Therefore, the inclusion of pressure homogenization degraded and disrupted the MFGM by promoting the interfacial adsorption of the milk protein (more casein) into the newly created MFGM surface to form strong linkages, specifically in HRM and HPM’s final viscosities [59], which became highly resistant to flow, adequately stabilizing the set yogurts [60].

### 3.5. Microstructure of Set Yogurts

**Figure 6:**
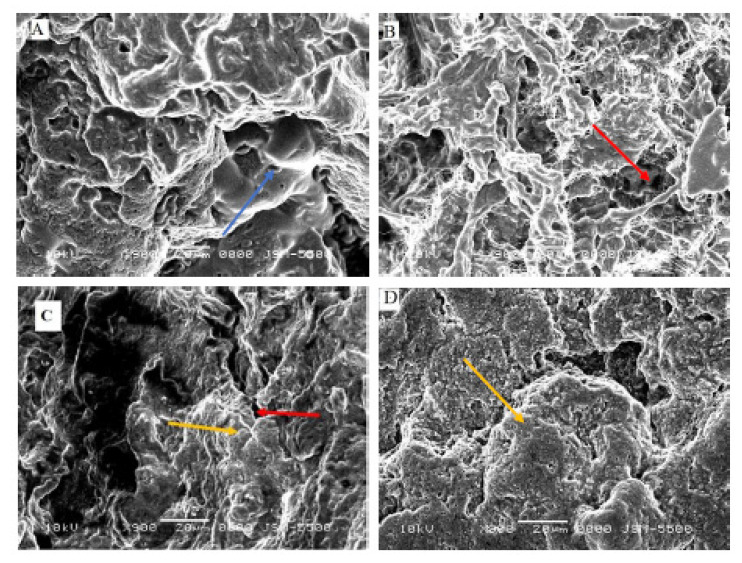
The SEM images of set yogurts produced from homogenized and non-homogenized raw milk and processed milk combined with 4% Lacprodan^®^PL20. (**A**) Non-homogenized raw milk (NRM control sample). (**B**) Non-homogenized processed milk (NPM). (**C**) Homogenized raw milk (HRM). (**D**): Homogenized processed milk (HPM). The red, yellow, and blue arrows indicate the holes, surface interactions, and MFGM fragments, respectively. The scale bar is 20 µm.

The microstructures were visualized under SEM to elucidate the basic interactions of the MFGM components with milk fractions (Figure 6). Homogenization significantly changed the physical and textural attributes, as reflected in the microstructures of the set yogurt prepared. In Figure 6A,B, heat treatment systemically enhanced the linkages between casein and whey proteins with MFGM through sulfhydryl groups [61], showing small holes within the compartment. The presence of large MFGM fragments was seen in the microstructure of set yogurt produced from non-homogenized raw milk (NRM) due to certain small fragments that merged together to form aggregates. The NPM microstructure was fluffy and less dense as a result of heat treatment (milk pasteurization) before adding MFGM and a lack of pressure homogenization, which led to limited interaction between milk proteins and the MFGM. In support of these changes, the firmness and apparent viscosity of NPM were very low (Table 2 and Table 3), pointing to a weak interaction within the system.

On the other hand, homogenization clearly bridged the gaps within HRM (Figure 6C), through destabilization of the MFGM fragments into more surface active agents that promote protein interaction [62,63]. The structure of HPM (Figure 6D) was moderately dense with wide cracks and small spots, closely related to NRM (some results of physicochemical properties were similar). Perhaps resistance of some MFG to disruption by homogenization resulted in small fat globules surrounded by MFGM, i.e., clusters of fat globules with partly disrupted MFGM or triacylglycerides (TAGs) filling voids in the protein matrix [2,7,64]. The presence of pore holes in NPM and HPM reduced the water-holding capacity, thus demonstrating no significant difference compared to NRM (Figure 4). Moreover, the structure of set yogurt produced from homogenized raw milk (HRM) with MFGM was densely linked together with little or no separation, which totally differs from NRM; these changes showed some significant gain in the water-holding capacity, firmness, and apparent viscosity (Figure 4, Table 2 and Table 3). Changes in the type of milk (raw versus processed) and process conditions (homogenization) primarily influenced the microstructures of set yogurt with explicit characteristics. In summary, supplementing set yogurts with the MFGM and applying a specific homogenization process could improve the microstructures and avoid some structural and textural defects, therefore, changing consumer’s perceptions and improving the quality of dairy products.

## 4. Conclusions

The increase in the surface interaction of MFGM with milk proteins due to homogenization contributed to the development of fine structures and changes in the composition as well as textural improvement in the set yogurts produced. As expected, homogenized set yogurts were significantly better than non-homogenized even though MFGM was added to all samples. The use of processed milk to produce set yogurts reduced the firmness, water-holding capacity, and viscosity of set yogurts compared to raw milk, therefore, limiting the interaction with the MFGM fragment. The viscosity and water-holding capacity were found to increase because of the MFGM supplementation and homogenization process. Future studies should focus on the novel processing technique’s impact on MFGM interaction, distribution, and characterization with plant-based dairy proteins, as well as the kinetics of MFGM quality changes during non-thermal processing.

## Figures and Tables

**Table 1:** Composition of set yogurts produced from raw milk or processed milk combined with Lacprodan^®^PL20 on a dry-matter basis expressed as (g/100 g).

Set Yogurts	TS	TS Non-Fat	Protein	Fat	Ash	Lactose
NRM	18.62 ± 0.18 ^AB^	14.37 ± 0.38 ^A^	37.17 ± 0.19 ^A^	22.84 ± 1.39 ^B^	7.09 ± 0.30 ^AB^	32.90 ± 1.59 ^B^
NPM	17.49 ± 0.22 ^B^	13.01 ± 0.38 ^B^	36.03 ± 0.71 ^A^	25.64 ± 1.34 ^AB^	7.19 ± 0.18 ^A^	31.14 ± 1.82 ^B^
HRM	19.48 ± 1.05 ^A^	14.06 ± 1.11 ^AB^	33.90 ± 2.88 ^AB^	27.91 ± 2.41 ^A^	6.67 ± 0.26 ^B^	31.52 ± 4.70 ^B^
HPM	18.51 ± 0.04 ^AB^	14.23 ± 0.17 ^AB^	30.48 ± 1.33 ^B^	23.11 ± 1.01 ^B^	6.78 ± 0.24 ^AB^	39.64 ± 1.10 ^A^

The data are expressed as means ± standard deviation of two replicates. The different superscripts represent the significant differences among the set yogurts (Tukey’s test, *p* < 0.05). TS: Total solids, NPM: Non-homogenized processed milk, NRM: Non-homogenized raw milk, HRM: Homogenized raw milk, HPM: Homogenized processed milk.

**Table 2:** Firmness of set yogurt produced with an enriched MFGM.

Set Yogurts	Firmness (g)
NRM	28.00 ± 1.41 ^AB^
NPM	18.50 ± 2.12 ^B^
HRM	38.50 ± 2.12 ^A^
HPM	23.50 ± 3.54 ^B^

The data are expressed as means ± standard deviation of two replicates. The different superscripts represent the significant differences among the set yogurts according to the results of Tukey’s test, *p* < 0.05. NPM: Non-homogenized processed milk, NRM: Non-homogenized raw milk, HRM: Homogenized raw milk, HPM: Homogenized processed milk.

**Table 3:** The initial and final apparent viscosities of set yogurts combined with enriched MFGM and their relationship with loss of structure after a two-stage pressure homogenization process.

Apparent Viscosity vs. Time (Pa.s) at 0.5 s^−1^			
Set Yogurts	Initial Viscosity $\left(\eta_{0}\right)$	Final Viscosity $\left(\eta_{f}\right)$	Loss of Structure (%)
NRM	15.83 ± 0.83 ^AB^	5.89 ± 0.16 ^B^	62.72 ± 2.95 ^A^
HRM	18.70 ± 1.61 ^A^	10.49 ± 1.91 ^A^	44.12 ± 5.40 ^B^
NPM	7.64 ± 0.26 ^C^	4.58 ± 0.20 ^B^	42.52 ± 8.18 ^B^
HPM	13.34 ± 0.14 ^B^	7.18 ± 0.48 ^AB^	46.20 ± 3.03 ^B^

The data are expressed as the means ± standard deviation of two replicates. The different superscripts represent the significant differences among the set yogurts according to the results of Tukey’s test, *p* < 0.05. NPM: Non-homogenized processed milk, NRM: Non-homogenized raw milk, HRM: Homogenized raw milk, HPM: Homogenized processed milk.

## Data Availability

The datasets generated during and/or analyzed during the current study are not publicly available due to a research policy but are available from the corresponding author on reasonable request.
